# Supplementary material for: The Effect of Different Substrates on the Morphological Features and Polyols Production of Endomyces magnusii Yeast during Long-Lasting Cultivation
Source: Microorganisms. 2022 Aug 25;10(9):1709. doi: 10.3390/microorganisms10091709 (PMC9506286; doi:10.3390/microorganisms10091709)
Supplement: Supplementary file 1 [file microorganisms-10-01709-s001.zip › microorganisms-1822060-supplementary.pdf]

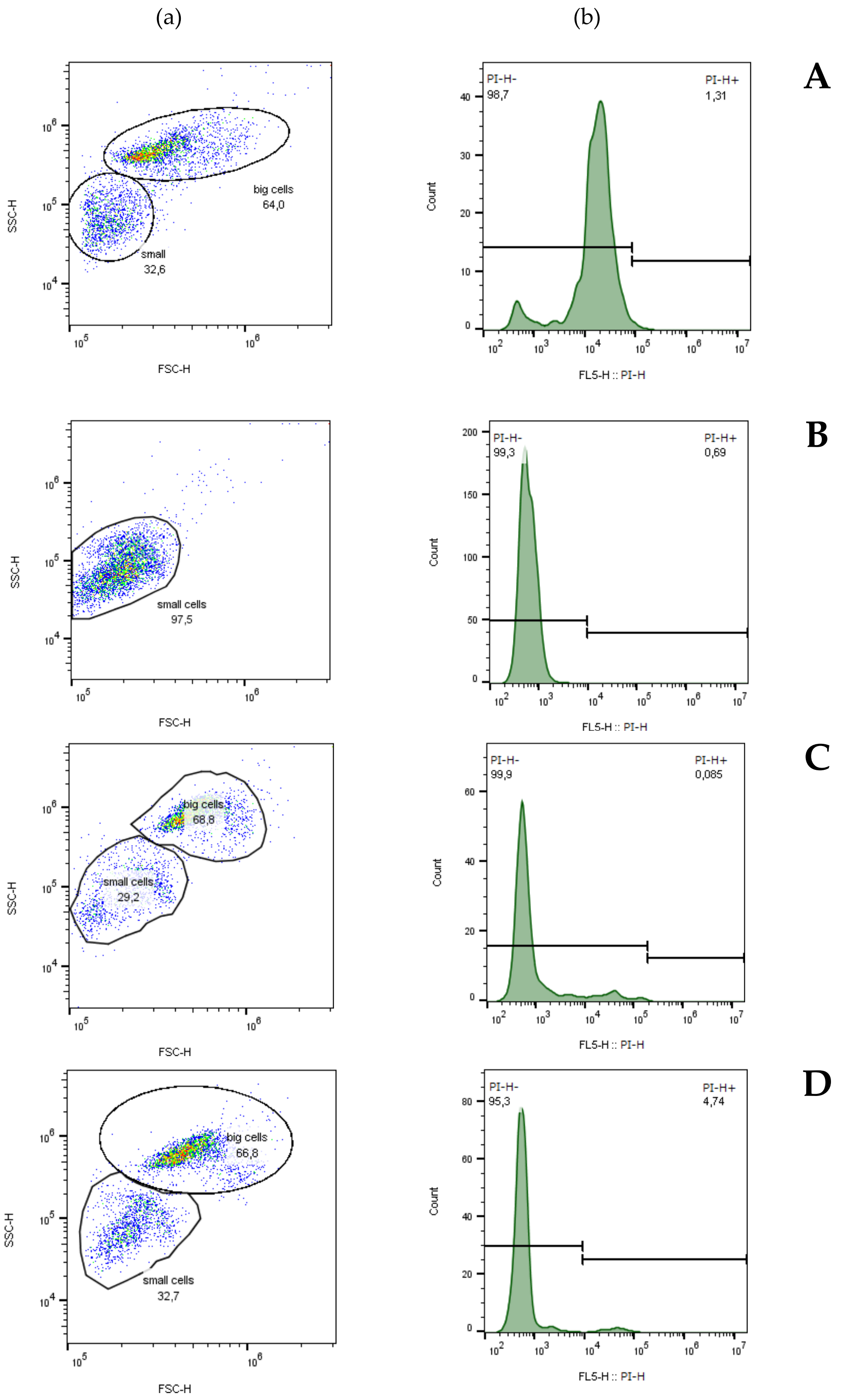

Figure S1. Distribution histograms of the *E. magnusii* yeast at the logarithmic growth stage (18 hours of cultivation) using flow cytometry. A - glycerol; B - 2% glucose; C - 0.5% glucose; D - 0.2% glucose; a - size distribution of the cell population; b – distribution of live and dead cells in the subpopulation of small cells. The cells are stained with PI (see Materials and Methods for details). Notations: left panel shows the cell size distribution. x-axis (FSC-H) - forward scatter, y-axis (SSC-H) - side scatter. Right panel shows the cytogram of PI/FITC-PNA staining. The cells can be divided into vital / non-live ones according to their green or red PI fluorescence. The x-axis (FL5-H) is the PI fluorescence intensity; the y-axis is the number of the cells tested.

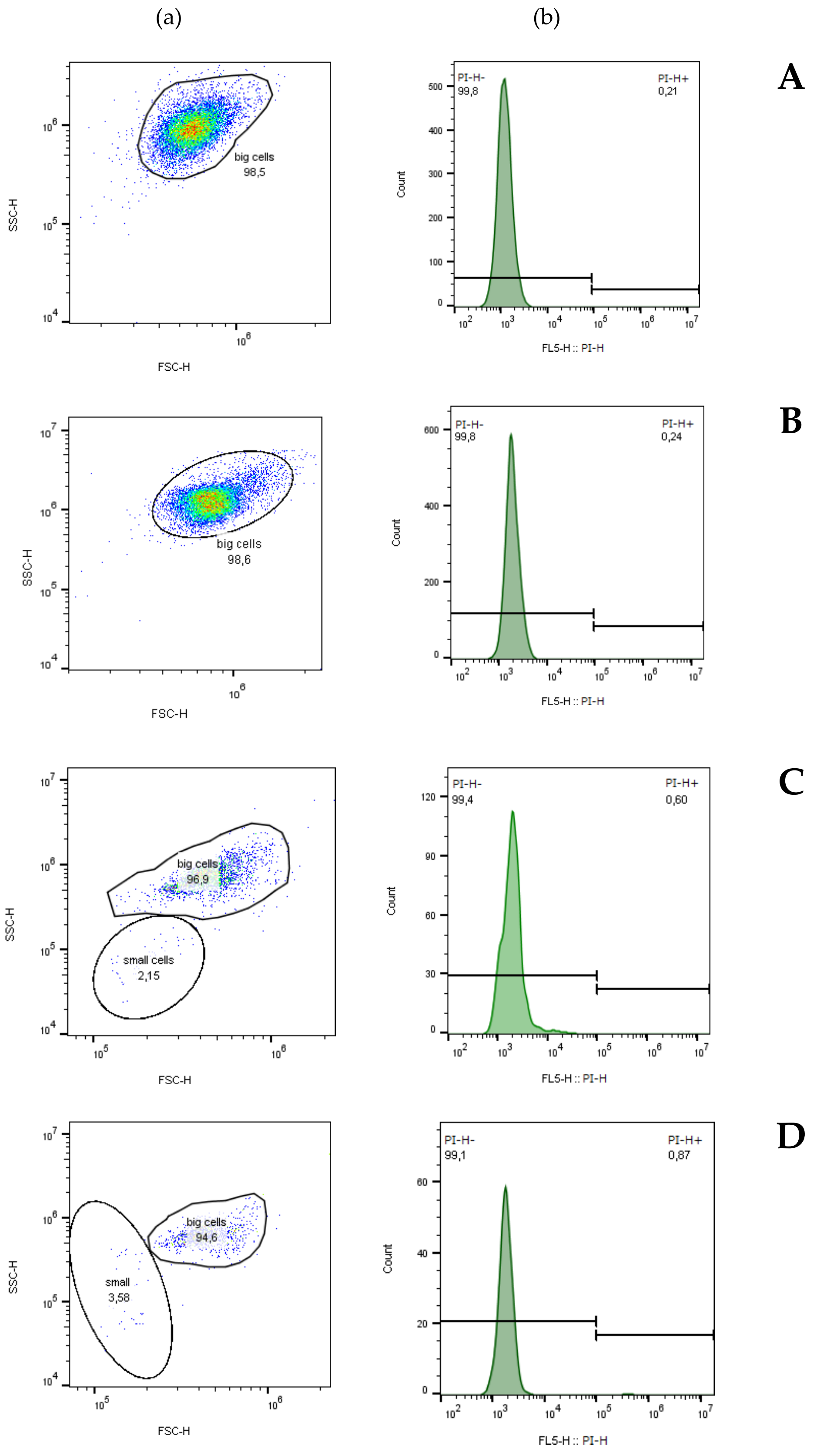

Figure S2. Distribution of the *E. magnusii* yeast at the stationary growth stage (2 weeks of cultivation) using flow cytometry. A - glycerol; B - 2% glucose; C - 0.5% glucose; D - 0.2% glucose; a - size distribution of the cell population; b – distribution of live and dead cells in the subpopulation of small cells. Cells are stained with PI (see Materials and Methods for details). Notations: left panel shows the cell size distribution. x-axis (FSC-H) - forward scatter, y-axis (SSC-H) - side scatter. Right panel shows the cytogram of PI/FITC-PNA staining. The cells can be divided into vital/non-live ones according to their green or red PI fluorescence. The x-axis (FL5-H) is the PI fluorescence intensity; the y-axis is the number of the cells tested.

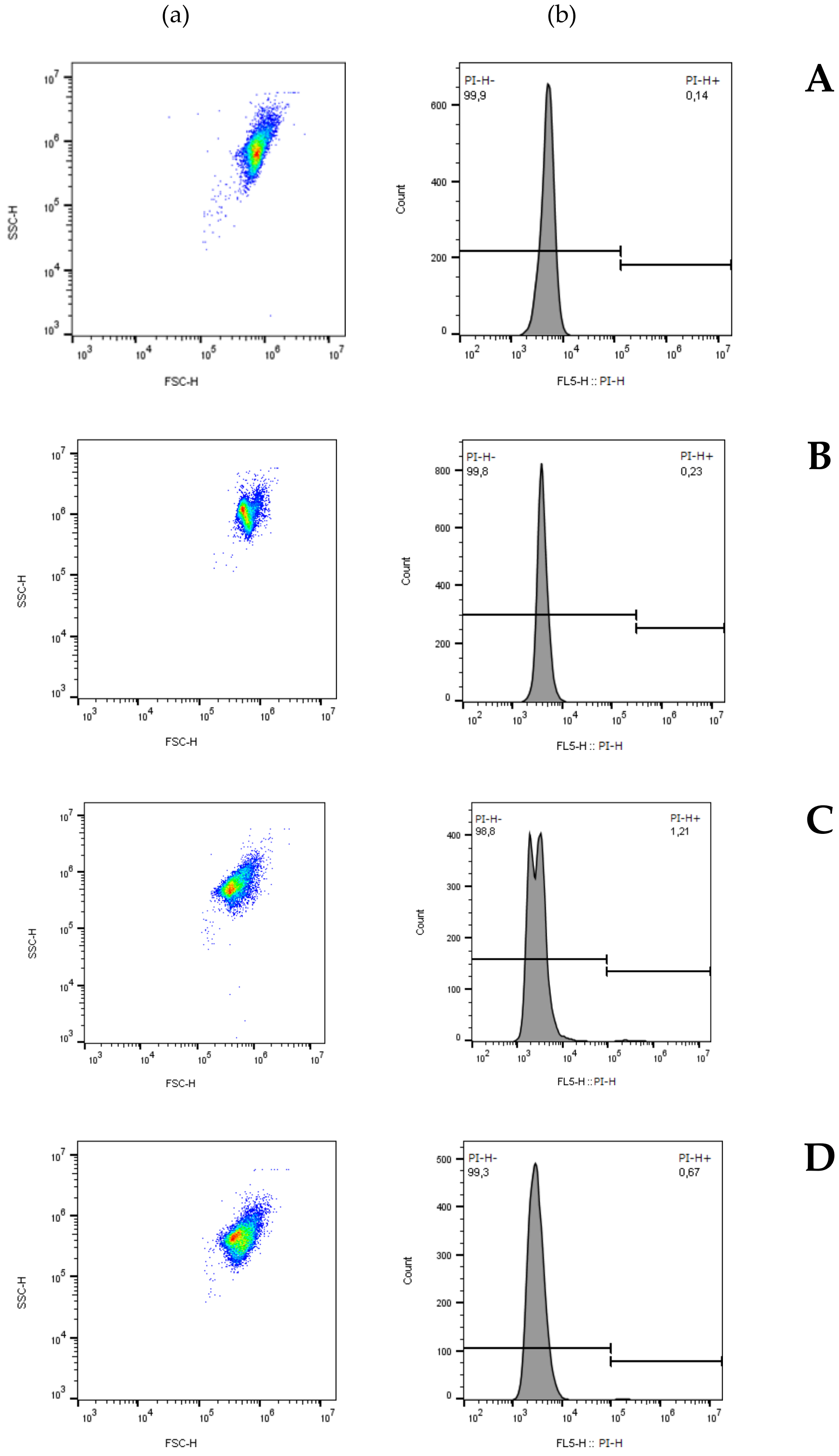

Figure S3. Distribution of the *E. magnusii* yeast at the stationary growth stage (4 weeks of cultivation) using flow cytometry. A - glycerol; B - 2% glucose; C - 0.5% glucose; D - 0.2% glucose; a - size distribution of the cell population; b – distribution of live and dead cells in the subpopulation of small cells. Cells are stained with PI (see Materials and Methods). Notations: left panel shows the cell size distribution. x-axis (FSC-H) - forward scatter, y-axis (SSC-H) - side scatter. Right panel shows the cytogram of PI/FITC-PNA staining. The cells can be divided into vital/non-live ones according to their green or red PI fluorescence. The x-axis (FL5-H) is the PI fluorescence intensity; the y-axis is the number of the cells tested.

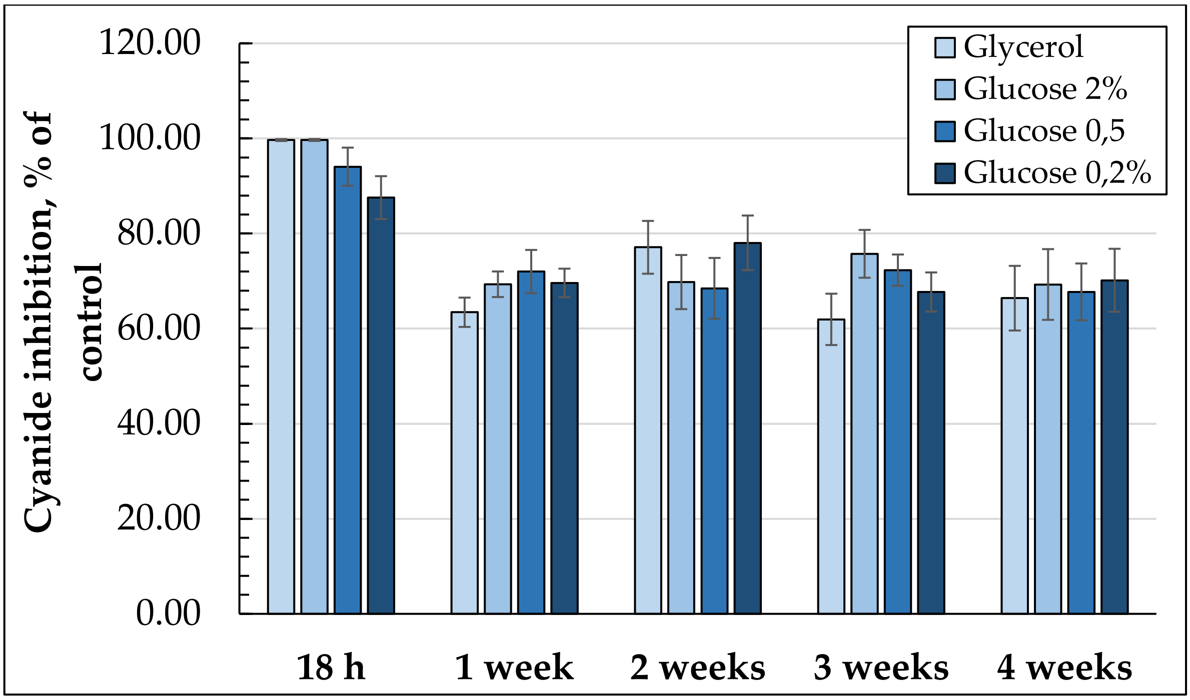

Figure S4. Respiratory activity of the *E. magnusii* yeasts with 4 mM KCN during long-lasting cultivation using different substrates.
